# Patient-Reported Outcome Measures (PROMS) in Lymphoma

**DOI:** 10.3390/curroncol32050265

**Published:** 2025-05-01

**Authors:** Neha Akkad, Christopher R. Flowers

**Affiliations:** 1 Hematology and Medical Oncology Fellowship Program, UT MD Anderson Cancer Center, Houston, TX 77030, USA; 2 Department of Lymphoma and Myeloma, UT MD Anderson Cancer Center, Houston, TX 77030, USA; crflowers@mdanderson.org

**Keywords:** lymphoma, patient-reported outcomes, quality of life

## Abstract

Patient-reported outcome measures (PROMs) are often used to evaluate the impact of treatment and clinical decisions on the patient experience for patients with lymphoma. Regulatory agencies have provided guidance on the use of PROMs for patient-focused drug development. Though PROMs are increasingly utilized, the way in which they are used, analyzed, and reported is heterogeneous. This systematic evidence-based review will focus on how PROMs are currently used for patients with lymphoma, what domains PROMs measure, their clinical significance, links to clinical outcomes, and what gaps need to be filled to better incorporate PROMs as endpoints in clinical trials and clinical decision-making.

## 1. Introduction

Patient-reported outcome measures (PROMs) are becoming more widely used in oncology and drug development in the modern era, including in patients with lymphoma. PROMs are standardized and validated survey tools that assess health outcomes as reported by patients themselves without interpretation from clinicians. PROMs can cover several domains, including general health, functional status, and quality of life (QoL), among others. Patient-reported outcomes (PROs) and their assessment have become more prevalent as studies have shown that physicians often underreport symptom burden as compared to patients, and that there is not always correlation between the physician assessment of the patient experience and their own lived experience [1,2,3,4,5,6]. As such, PROMs are increasingly used in patients with lymphoma to assess the patient experience in a way that other modalities cannot. PROMs assess how the patient experience changes in parallel to treatment response and how new drugs impact the patient experience, including QoL, symptom burden, and functional status. In light of this, the International Conference on Harmonization (ICH) and the Food and Drug Administration (FDA) have acknowledged the use of PROs in the development of oncology drugs. The FDA has also provided guidance on patient-focused drug development [7,8,9]. Novel therapies are often approved in oncology based on safety and efficacy, frequently using response and survival as endpoints. However, although we know that PROs are important to consider when treating patients with lymphoma, they are seldom incorporated into the approval process, even if PRO data were collected in the study evaluating the drug.

An analysis of the use of PROs in the approval of novel oncology drugs from 2017–2022 showed that PROs did not play a significant role in influencing the regulatory review of the evaluated drugs [10]. This analysis evaluated 67 novel oncology products, including 9 for lymphoma, and showed that 45.7% included PROs in the FDA approval documents; however, only 2 products included it in the original label. Details of the PRO data were often not disclosed due to the exploratory nature of the data, issues with trial design, PROM selection, statistical analysis, and PRO data collection [10]. This review will therefore focus on how PROs are currently used in patients with lymphoma, what themes we focus on, their clinical significance, relationships with clinical outcomes, and what gaps need to be filled in order to better incorporate PROMs as endpoints in clinical trials and clinical decision-making.

## 2. Methods

This systematic review was performed in accordance with the PRISMA guidelines [11,12] (Supplementary Figure S1). We searched PubMed from 2015–2025 to identify articles published regarding PROMs involving patients with lymphoma. Articles were removed if they were focused on a pediatric patient population or an unrelated topic or if lymphoma patients were included but not the focus of the study. Additional articles were included for certain topics that required more detail. We searched Clinicaltrials.gov from 2020–2024 to identify clinical trials involving patients with lymphoma that included PROMs and were phase 2 or 3, interventional, and carried out in the United States. Reference lists were cross-checked for relevant citations and duplications. We used the MeSH terms “Lymphoma” and “Patient Reported Outcomes.” The second reviewer provided final confirmation of all articles after initial review. For each study, the following data were collected on eligible publications: type of publication, type of study, and application of PROMs. After review and exclusion of articles and abstraction of data, we concluded that the limited number of articles and heterogeneity of clinical outcome measures were insufficient for a meaningful meta-analysis. All studies were, therefore, summarized descriptively as a systematic review. This review was registered in PROSPERO (ID1007936).

## 3. Current Role of PROMs in Prospective Clinical Trials

PROMs are often administered to patients as part of clinical trials evaluating the safety and efficacy of novel drugs and treatment modalities for patients with lymphoma. One recently published systematic review evaluating 90 phase 3 randomized controlled trials evaluating therapies for hematologic malignancies found that 73% included PROs as an endpoint, but only one as a primary endpoint; 56% included a PRO measurement as a secondary endpoint, 17% as an exploratory endpoint, and 27% did not include PROs as a prespecified endpoint. Of the 66 trials that included a PRO measure, 26 (39%) trials reported these data in the primary publication. A total of 29 trials in this review included lymphoma patients, of which 18 trials included a PRO as an endpoint, and only 3 trials reported these data in the primary publication. Almost all trials used PROMs focused on general QoL and symptom-specific metrics [13]. Related results were presented at the European Hematology Association’s (EHA) annual meeting in 2023 for patients with Hodgkin lymphoma (HL). Among 51 ongoing trials, 16 mentioned PRO assessment in their registry entries, protocol, or publications as a secondary or exploratory outcome, and 26% of published trials reported on PROs. The most referenced PROMs used were the European Organization for Research and Treatment of Cancer (EORTC) QoL questionnaire (EORTC-QLQ-C30; 12 studies), Euroqol (EQ-5D; 3 studies), and Functional Assessment of Cancer Therapy (FACT) Neurotoxicity (2 studies). Notably, none of the trials used the EORTC QLQ-HL27 or FACT-Lymphoma (FACT-Lym) disease-specific PROMs [14,15]. These studies highlight that while PROs are being used in clinical trials, they are not standardized and do not necessarily use disease-specific measures. The results of these metrics are often not reported in the primary publication for the associated clinical trial. A summary of clinical trials for lymphoma patients that report PROs used in their study plan is shown in Table 1 and Table 2. 2020–2024 was selected as the timeframe, as that follows the year that the FDA acknowledged PRO use in oncology drug development. There were 53 total phase 2 or phase 3 interventional trials that included PRO measurements that involved patients with lymphoma and were carried out in the United States. The most common lymphoma subtypes were aggressive lymphoma/large B-cell lymphoma (LBCL) and indolent lymphoma (iNHL), including follicular lymphoma (FL) and marginal zone lymphoma (MZL). There was no trend observed in increasing the use of PROMs over time. PROMs were included if they were used in more than one trial.

The five most commonly used PROMs, as noted in Table 2, were EORTC-QLQ-C30, EQ-5D-5L, FACT-Lym, FACT-G, and PRO-CTCAE. The most commonly focused-on domain was QoL. The EORTC-QLQ-C30 was first developed in 1993 and is validated to measure different aspects of QoL in cancer patients. It was shown to be valid in patients receiving palliative radiation, could detect the effect of radiotherapy, and was reliable in all scales except for role functioning [16]. EQ-5D-5L is a generic instrument to measure health-related QoL, which consists of a visual analog scale, as well as a descriptive assessment with five dimensions: mobility, self-care, usual activities, pain/discomfort, and anxiety/depression [17]. FACT-G is a general cancer QoL assessment. It was shown to demonstrate sensitivity to change over time, and coefficients of reliability and validity were high in a study, including mixed cancer diagnoses [18]. FACT-Lym was then developed to assess HRQoL in lymphoma patients; specifically, it was validated in a study, including 60% indolent lymphoma patients and 85% of patients on treatment. It was shown to have test-retest reliability (0.82) and internal consistency. It was correlated with SF-36 to show validity and was developed to be used as an endpoint in clinical trials [29]. The PRO-CTCAE is a 124-item library built as a companion to the common terminology criteria for adverse events (CTCAE). These items can be selected and combined based on the clinical study they are being used for [19]. Links to these PROMs can be found in Supplementary Table S1.

More recent clinical trials, especially clinical trials evaluating the safety and efficacy of chimeric antigen receptor T-cell (CAR-T) therapy, have focused more on PROs. Given CAR-T is intended as a one-time therapy and has been compared to standard of care and stem cell transplant (SCT) and PROs are feasible to collect, it is important to know if there are acute or chronic impacts on the patient experience that may not be apparent when analyzing safety and efficacy [30]. One trial that evaluated QoL was Zuma-7, which compared axicaptagene ciloleucel (Axi-cel) versus standard of care (SOC) in second-line (2L) relapsed refractory (R/R) diffuse large B-cell lymphoma (DLBCL). Among 296 patients evaluated using EORTC QLQ-C30 physical functioning, global health status/QoL, and EQ-5D-5L, this study showed statistically significantly higher scores in patients receiving axi-cel compared to SOC for QLQ-C30 global health status/QoL and EQ-5D-5L visual analog scale (VAS) at multiple time points [31]. Similarly, a single-arm phase 2 study of lisocaptagene maraleucel (liso-cel) in R/R DLBCL reported the impact on PROs using EORTC QLQ-C30, FACT-Lym, and EQ-5D-5L. This study showed that there was an overall mean improvement in scores from baseline through day 545 for the EORTC QLQ-C30 fatigue, pain, and appetite loss, as well as the FACT-Lym and euroqol visual analog scale (EQ-VAS). Within patient analysis, scores were maintained in the same patient across multiple time points, indicating that liso-cel did not worsen PROs and improved QoL in some domains [32]. Health-related QoL (HRQoL) was also evaluated in the phase 2 JULIET study, evaluating the safety and efficacy of tisagenlecleucel (tisa-cel) in R/R DLBCL. FACT-Lym and Short Form Health Survey (SF-36) were administered at baseline and months 3, 6, 12, and 18. This study found that patients who had a complete response (CR) or partial response (PR) had an improvement in FACT-Lym scores at all time points compared to baseline, and SF-36 scores showed improvement above the minimal clinically important differences on five of eight subscales [33]. Since each of these trials used different PROMs for assessment, it is challenging to make comparisons or to attribute common QoL effects across these studies.

More recently, bispecific antibodies have also become a treatment option in R/R DLBCL. A study evaluating epcoritamab, a CD20 × CD3 bispecific antibody, for R/R LBCL found that in 157 patients included, scores on both FACT-Lym and ED-5D-3L improved from baseline to cycle 9 and exceeded minimally important difference thresholds, especially in patients that responded to therapy [34]. These studies highlight the way PROs have been applied in CAR-T and bispecific antibody clinical trials. Similar survey tools were used across studies, namely EORTC-QLQ-C30, EQ-5D-5L, FACT-Lym, and SF-36, representing a combination of both general and disease-specific measures. In CAR-T trials, PROs were evaluated in the short-term setting, and it was found that PROs largely improved after CAR-T therapy. Additionally, it appears that better PRO scores were associated with treatment response in studies in which that analysis was performed. Further follow-up will be required to determine if there are unexpected long-term impacts on the patient experience and patient QoL as these patients continue to have long-term follow-up.

PROMs have also been used recently in other subtypes of lymphoma, including FL and cutaneous T-cell lymphoma (CTCL). The ROSEWOOD trial evaluated zanubrutinib plus obinutuzumab versus obinutuzumab alone in patients with FL who had at least two lines of prior therapy [35]. HRQoL was evaluated in this study using EORTC-QLQ-C30 and EQ-5D-5L. This study found that at 48 weeks, patients receiving zanubrutinib + obinutuzumab had improved role functioning and fatigue and nausea/vomiting compared to the obinutuzumab alone arm, but similar scores in the EQ-5D-5L VAS [36]. Given studies have shown that QoL declines with subsequent lines of therapy in patients with FL [37], clinical trials evaluating therapies in the R/R FL space should continue to evaluate PROs and impact on QoL so physicians can better select and sequence therapy in patients with heavily pre-treated FL. For patients with CTCL, mogamulizumab was compared to vorinostat in a study of 372 patients. QoL was measured using Skindex-29 and FACT-G. This study found that patients receiving mogamulizumab had an improvement in Skindex-29 scores in the symptoms (Cycles 3,5,7; *p* < 0.05) and functional subscales (Cycles 3,5; *p* < 0.05), respectively. Median time to worsening of the symptoms domain of the Skindex-29 was 27.4 months for mogamulizumab versus 6.6 months for vorinostat. They additionally found statistically significant improvement in physical well-being as measured by FACT-G in favor of mogamulizumab (Cycles 1,3,5; *p* < 0.05). These differences were more pronounced in patients with Sezary syndrome (SS) compared to patients with mycosis fungoides (MF) [38]. Brentuximab vedotin (BV) was also studied in patients with CD30+ relapsed CTCL. QoL was assessed in a complementary study using Skindex-29, FACT-G, and EQ-5D. This study found that there was a reduction in Skindex-29 symptom domain scores in patients who received BV versus physicians’ choice that exceeded the prespecified minimally important difference (MID), the difference was found to be −18.9 (MID 9.0–12.3). This difference remained true when evaluating only patients who developed peripheral neuropathy, which is of importance as neuropathy is a known adverse effect of BV. There was no difference found between groups in FACT-G and EQ-5D scores [39]. HRQoL was also reported on in the phase 2 study evaluating the safety and efficacy of lacutamab in patients with relapsed CTCL. It was found that there was a clinically meaningful decrease in itch (≥2 points on VAS-itch scale) from week 13 for patients with SS and week 37 for patients with MF. There were also improvements in Skindex-29 scores over time in both SS and MF [40]. These studies highlight that QoL can be evaluated with PROs and are easier to interpret with disease-specific measures, such as Skindex-29. These studies also highlight that though QoL is an important measure in this disease, in pivotal clinical trials for novel therapies in this space, QoL was not reported or partially reported in the primary publication leading to drug approval. These studies also emphasize that in CTCL in particular, a more disease-specific PROM than what currently exists would be useful both in clinical decision-making as well as to be used as an endpoint in clinical trials.

## 4. PROMs in Other Clinical Studies

Beyond their use in clinical trials, there are multiple areas in which PROMs have been used in studies for patients with lymphoma. Through our literature review, the most prominent use outside of prospective clinical trials for novel drugs has been when examining specific modalities of treatment and their effect on PROs and evaluating long-term treatment strategies for lymphomas, such as for indolent lymphoma, mantle cell lymphoma (MCL), chronic lymphocytic leukemia/small lymphocytic lymphoma (CLL/SLL), and CTCL.

### 4.1. Real-World Experiences Evaluating PROMs for Lymphoma Patients Undergoing Chemotherapy

PROMs are often used to help providers assess the potential risks and benefits of therapies and treatment modalities that either have not been or are not able to be compared head-to-head. One small study used PROMs to evaluate if there was a difference in QoL in 35 patients with lymphoma or myeloma who were treated in a clinical trial versus SOC and found that when evaluating QoL measured by FACT-G or perception of care, there was no difference between groups at 3 months or 12 months [41]. The authors, therefore, concluded that receiving treatment on a clinical trial would not necessarily have an impact on patient QoL or their perception of their quality of care. However, given the small and disparate sample size used in this study, it is difficult to draw any conclusions if this will apply to all lymphoma subtypes given the heterogeneity in treatments for different lymphoma subtypes and how that may impact patient QoL.

### 4.2. Real-World Experiences Evaluating PROMs for Lymphoma Patients Undergoing SCT or CAR-T

Beyond comparing clinical trials versus SOC, most notably, PROs have been a focus of studies evaluating patients with lymphoma receiving SCT versus CAR-T, as both remain standard of care options but have not been directly compared head-to-head in all lymphoma subtypes in which they are indicated as a treatment option. One large study was carried out in the United States of 980 patients with aggressive lymphoma to evaluate long-term PROs in patients who received an autologous-SCT compared to those that did not as measured by FACT-G, Functional Assessment of Chronic Illness Therapy (FACIT) fatigue scale, State-Trait Anxiety Inventory (STAI), and Profile of Mood States instruments with a numeric rating scale for overall QoL and spiritual QoL measured at 3 years and 6 years from lymphoma diagnosis. This study found there was no difference in the transplant versus no transplant group at 3 years (*p* = 0.64) and 6 years (*p* = 0.44) for FACT-G scores and no other clinically significant differences in any of the QoL instruments. The transplant group had lower scores for anxiety as measured by the STAI. The treatment that the no-transplant group received was not reported in this study [42]. A similar study was carried out in Norway that evaluated 271 patients who received high-dose chemotherapy with an autologous-SCT for lymphoma in order to evaluate the late side effects and effect on HRQoL. This study evaluated patients at a median of 8 years of follow-up and found that physical and mental health scores, as assessed by the SF-36, were lower in patients that had received a transplant as compared to age and sex-matched controls and that a high late symptom effect was significantly associated with worse scores for physical and mental health [43]. A third small study evaluated 23 patients receiving CAR-T cell therapy for HL and used Patient-Reported Outcomes Measurement Information System (PROMIS) global physical health, PROMIS global mental health, and PROMIS physical function to assess PROs. Overall symptom burden was assessed using National Cancer Institute (NCI) PRO-CTCAE to show that scores on these surveys and overall symptom burden were largely similar to pre-stem cell procurement and post-infusion [44]. Another study evaluated 120 patients receiving CAR-T for LBCL and evaluated QoL in the first year post-CAR-T. This study found that QoL, measured by PROMIS-29, was unchanged or improved in the year after treatment and that patients that did not respond had worse QoL scores [45]. These studies suggest that QoL largely remains intact over time in patients that were treated with a SCT and CAR-T in different lymphoma subtypes; however, patients with a higher symptom burden are at higher risk of having a negative impact on PROs and QoL. Therefore, targeting late symptoms could help improve QoL, and studying interventions that target physical and mental health, such as diet, exercise, smoking, etc., as well as early prevention and treatment of other co-morbidities, is of importance in this patient population. CAR-T cell therapy will require longer-term follow-up to elucidate if there are long-term impacts on PROs that are not evident in the acute or shorter-term setting, as this treatment modality has been FDA-approved more recently than SCT for the treatment of R/R lymphoma.

## 5. PROMs to Evaluate Longitudinal Outcomes

### 5.1. Indolent Lymphoma

Another area in which PROMs are often used is to evaluate how PROs change over time in subtypes of lymphoma that are often incurable. In this setting, the impact of treatment on the disease has to be weighed against the impact on the patient experience and QoL. This is true for FL, which is often not cured with standard treatment, and patients can live many years both on and off treatment. One study evaluated 253 patients with FL enrolled in the RESORT study that compared maintenance rituximab dosing versus re-treatment with rituximab dosing after induction rituximab in patients with low tumor burden FL to compare illness-related anxiety between groups, as measured by 22-item Impact of Event Scale-Revised (IES-R) as the primary endpoint. This study found that there was no significant difference in illness-related anxiety between groups and that HRQoL did not change significantly or differ between groups as measured by FACT-G [46]. A second smaller study evaluated a similar concept among 52 patients undergoing active surveillance for indolent lymphoma using FACT-Lym to evaluate QoL. This study showed that HRQoL was unchanged across multiple time points up to 3 years from diagnosis. Patients with indolent lymphoma on active surveillance were found to have higher scores in physical, functional, and social well-being but not emotional well-being compared to the general population norm scores [47]. There was no difference found for emotional well-being. This theme was again studied in a prospective study of 91 patients with any CD20+ NHL (majority DLBCL) that compared QoL in patients randomized to 2 years of R maintenance versus observation after standard treatment. This study found there was no difference in QoL between groups as measured by EORTC-QLQ-C30, EuroQol-5D, and EuroQol-5D VAS [48]. Taken together, these studies use PROMs to help evaluate if surveillance/maintenance treatment causes a negative impact on QoL compared to an observation strategy or if a watch-and-wait strategy increases anxiety or has other potentially patient-centered consequences that physicians may not be picking up on. Based on these studies, it appears that treatment with maintenance rituximab in various settings does not have a negative impact on PROs and that active surveillance does not increase patient anxiety or have a negative impact on HRQoL. Therefore, clinicians can employ the watch-and-wait strategy if clinically appropriate to avoid overtreatment in patients that may not need it, without having a significant impact on patient anxiety based on these studies.

Another way PROMs have been used in FL is to identify an unmet need for better treatments. One cross-sectional study evaluating QoL in 401 patients with FL across the United States and Europe used the EORTC QLQ-C30 to show that global health status/QoL and all functioning domains (excluding emotional functioning) worsened from first-line to third-line and beyond of treatment, with the worsening across lines of therapy in physical functioning, role functioning, and social functioning being statistically significant [37]. This study highlights that evaluating PROs is a way to evaluate an unmet need. Though FL has a very good 5-year OS of 89.9% (SEER) [49], QoL and functional status are still an issue for these patients, especially in later lines of therapy, meaning it should be evaluated in clinical trials that are studying novel therapies in that space, as was done in the ROSEWOOD trial described above.

### 5.2. Mantle Cell Lymphoma

Patients with MCL are living longer and often receive long-term, continuous therapies, especially in the relapsed setting. However, studies evaluating PROs for both limited and continuous treatments are limited. The BRIGHT study, a phase 3 randomized non-inferiority trial comparing bendamustine and rituximab versus R-CHOP/R-CVP in untreated indolent NHL and MCL, measured HRQoL with the EORTC-QLQ-C30. This study found that patients randomized to the BR arm had improved cognitive functioning, physical functioning, social functioning, emotional functioning, and global health status for at least one time point [50]. A study complementary to the SPRINT trial, which evaluated the safety and efficacy of lenalidomide versus investigator’s choice, found that in 254 patients with R/R MCL, HRQoL measured by the EORTC-QLQ-C30 demonstrated similar QoL scores between groups and a statistically significant improvement in QoL in the lenalidomide group compared to investigator’s choice for the physical functioning domain (24% vs. 8%; *p* = 0.003) and pain domain (29% vs. 18%; *p* = 0.047) [51]. A third trial comparing ibrutinib to temsirolimus also evaluated HRQoL using FACT-Lym and EQ-5D-5L. This study showed that 66% of patients receiving ibrutinib versus 48% of patients receiving temsirolimus had a clinically meaningful improvement in FACT-Lym scores, and patients receiving ibrutinib had improved EQ-5D-5L scores compared to baseline that was statistically significantly different from patients receiving temsirolimus for the first two cycles of treatment [52]. These studies highlight that long-term continuous therapies may not have a negative impact on patient QoL.

With the increasing use of continuous therapies for MCL in the modern era, such as bruton’s tyrosine kinase inhibitors (BTKi), it remains unclear if there are different impacts on QoL for different BTKis, as they have never been compared head-to-head. A recent study evaluating pirtobrutinib, a non-covalent BTKi, in 124 patients with R/R MCL evaluated patient-reported symptoms using 13 EORTC Item Library items and fatigue using 6 EORTC Item Library items. Most patients had either stable or clinically meaningful improved outcomes for each of these domains, including physical function (>80% of patients), QoL (>80%), MCL symptoms (>75%), and fatigue (>65%). However, compliance with surveys declined with each cycle [53]. Given there have not been head-to-head trials proving superiority of one continuous treatment over another, the impact of treatment on PROs may help physicians select therapy as they can take into account how each BTKi will influence their specific patient’s experience and thus is important to continue to include PROMs in trials evaluating therapies in this setting.

### 5.3. Cutaneous T-Cell Lymphoma (CTCL)

CTCL is another lymphoma subtype in which PROs and QoL are important components of clinical decision-making. CTCL has a detrimental impact on patient QoL, even compared to other malignancies and skin diseases, has a long duration of treatment, and is incurable without a SCT [54,55]. As such, clinical trials evaluating novel therapies in advanced stage disease have used PROMs to evaluate the impact of treatment on the patient experience. For example, the two FDA-approved systemic therapies for advanced stage CTCL, mogamulizumab and BV, reported on QoL, as was described above. In addition, a real-world study evaluated the impact of mogamulizumab on patient QoL in a small prospective study of 13 patients with erythrodermic CTCL. This study found that 36% of patients had an improvement in the overall Skindex-29 score, the Mental Component Score improved from 31 to 38, median VAS itch score improved from 8 to 3, and 7 of 13 patients were “very satisfied” with their treatment. Itch has been shown to be highly correlated with QoL in this disease, therefore showing mogamulizumab has a positive impact on patient QoL [56,57]. Given CTCL is a rare disease and treatment is heterogeneous, future studies should continue to focus on PROs in patients receiving treatment to further elucidate the real-world impact of treatment on PROs, especially QoL.

## 6. PROMs and Association with Clinical Outcomes

PROs have also been studied to assess their associations with clinical outcomes, such as response to therapy and survival for patients with lymphoma. One study evaluated 701 patients with aggressive lymphoma enrolled in the Molecular Epidemiology Resource of the University of Iowa/Mayo Clinic Lymphoma Specialized Program of Research Excellence and found that baseline QoL was independently predictive of OS after adjusting for IPI and lymphoma subtype. This study used the FACT-G Version 4 to measure QoL and a single-item measurement, Linear Analogue Scale (LASA), to measure overall and spiritual QoL. This study found that all baseline QoL measures, with the exception of emotional well-being (a subscale of FACT-G), were significantly associated with OS (*p* < 0.04), of which all but LASA spiritual remained significant after adjusting for IPI and NHL subtype. Patients with a low QoL score (overall QoL ≤ 50) had a median OS of 92 months compared to 121 months for patients with high QoL scores of >50 (*p* = 0.0004) [58]. One smaller study also supports this finding. This study evaluating 236 patients with DLBCL receiving R-CHOP found poor QoL measured by the EORTC QLQ-C30 to be associated with early treatment discontinuation [59]. This concept has also been shown in a meta-analysis of cancer patients in general [60]. These studies highlight that QoL can be associated with clinical outcomes, including OS and treatment discontinuation, which indicates that QoL should both be studied as an endpoint in clinical trials and taken into account when assessing patient’s clinical response and overall clinical status, as it may impact prognosis.

Another study evaluated the prognostic value of pretreatment PROMs in 1239 patients with DLBCL receiving treatment on the GOYA trial [61]. This study used EORTC QLQ-C30 and FACT–Lym surveys and used a Cox regression analyses of OS and progression-free survival (PFS) to identify PRO scales with high prognostic value and found that the FACT-Lym and EORTC QLQ-C30 physical functioning, global health status/quality of life (QoL), and fatigue retained significant prognostic value for OS and PFS after adjusting for multiple clinical variables (IPI, cell of origin, *BCL2* status, and total metabolic tumor volume) [61]. A second multivariable analysis of patients from the GOYA trial also found that after adjusting for disease risk at diagnosis, cell of origin, *BCL2* status, total metabolic tumor volume, and baseline PRO, measured by EORTC QLQ-C30, every 10-point increase in emotional functioning from baseline to C3D1 was associated with an 11% lower risk of progression or death (HR, 0.89; 97.5% CI, 0.78 to 1; *p  =*  0.02) and a 12% lower risk of death (HR, 0.88; 97.5% CI, 0.76 to 1.01; *p*  =  0.04). When FACT-Lym was used, every 1-point increase (worsening) in lumps or swelling symptoms, from baseline to C3D1 was associated with a 27% higher risk of disease progression or death (HR, 1.27; 97.5% CI, 1.02 to 1.58; *p*  =  0.01) and a 29% higher risk of death (HR, 1.29; 97.5% CI, 1.01 to 1.65; *p*  =  0.02), and with every 1-point increase in fever symptoms from baseline to C3D1 was associated with a 41% higher risk of death (HR, 1.41; 97.5% CI, 1.07 to 1.88; *p*  =  0.01) [62]. These studies suggest that patient QoL and symptoms are associated with survival in certain domains, OS more so than PFS, indicating that PROs likely do have some predictive value for clinical outcomes; however, it remains unclear at this time how these should be taken into account in a clinical setting.

## 7. Successful Implementation of PROMs for Patients in Hematologic Cancers

Myelofibrosis provides a clear example of a disease that has successfully implemented PROMs to measure symptom burden and QoL that have been validated over time in this specific patient population [63,64,65,66]. Most recently, the MFSAF TSS v4 (Myelofibrosis Symptom Assessment Form Total Symptom Score version 4) was validated in the phase 3 MOMENTUM study, which evaluated momelotonib versus danazol in patients with symptomatic myelofibrosis [67]. This study used MFSAF TSS v4 response at week 24 as the primary endpoint. This study showed a statistically significant decrease in MFSAF TSS in the momelotinib group compared to the danazol group (decreased score indicating improved QoL) (25% vs. 9% [95% CI 6–26]; *p* = 0·0095) [67]. These results led to the FDA approval of momelotinib for symptomatic myelofibrosis in 2023. This study highlights that in certain diseases in which there is high symptom burden and impact on QoL, as well as a potentially indolent course such as myelofibrosis and CTCL, clinical trials with endpoints focused on PROs are more clinically meaningful. The approval of momelotinib using MFSAF TSS also highlights the need for rigorously validated, disease-specific PROMs, especially in diseases with unique symptoms, such as CTCL.

## 8. Where Challenges Exist for Using PROMs and How We Can Improve

Though the importance of PROs has become evident in the modern era and PROMs are becoming increasingly incorporated both into clinical trials and observational clinical studies, the ideal use, tools, analysis, and reporting have remained challenging and therefore is not often taken into account for drug approval for patients with lymphoma. Part of the reason for this is because it is difficult to define QoL as an endpoint and the duration has to be chosen and interpreted carefully. For example, acute effects on QoL can be appropriate in clinical trials to evaluate the impact of a drug on QoL; however, long-term effects are more appropriate for indolent diseases or to assess long-term effects of one-time treatments, such as CAR-T or SCT [68].

To try and quantify the impact of a treatment on the patient experience, time to deterioration (TTD) has been used as an endpoint in oncology clinical trials to infer QoL and PROs. These can be defined as time to when a patient reports a symptom or functional decline. However, TTD has been shown to not be the best measure to infer QoL, as it is often defined differently in different trials and intercurrent events (death, progression, and discontinuation for other reasons) are not handled uniformly. One opinion piece published in *Lancet Oncology* in 2022 suggested, rather than using TTD, to instead use prespecified PRO analyses that evaluate comparative benefit in symptoms or functioning. Additionally, time points chosen should be clinically relevant and make sense in the context of the clinical question being answered [69]. Overall, this highlights that when PROMs are incorporated into studies, clinical trials or not, their analysis must be carefully prespecified, survey tools should be relevant to the clinical context, and their reporting should be pre-planned.

Additionally, there are particular lymphoma subtypes in which PROs should be taken into account more so than others due to the natural history of the disease and the relevance that PROs and QoL have on these particular patients. For example, in MCL, in the modern era, some patients can be treated with continuous BTK inhibitors; however, there are multiple options, and they have not been compared head-to-head. In this setting, it would be important to know their comparative long-term effect on QoL, as these patients will be on long-term treatment, and this impact will assist physicians in making treatment decisions in a patient-centered manner. In CLL/SLL, it is also important to evaluate QoL, as some patients will be on surveillance while others will be on treatment. It is, therefore, important to know what PROs are important to both CLL and MCL patients. One mixed-methods study attempted to answer this question by selecting items from the EORTC Item Library to form a basis for measuring CLL/SLL and MCL disease-related symptoms [70]. Interviews were completed with 57 patients (30 MCL, 27 CLL/SLL). Disease-related symptoms were then organized in a structured conceptual model, which was mapped to item sets from the EORTC Item Library. They found the symptom domains that were most important to CLL/SLL and MCL patients included swollen lymph nodes, B symptoms, abdominal issues, pain, fatigue, subjective cognitive impairment, anemia-related symptoms, bleeding, infection, and other issues (appetite loss, temperature fluctuation, rash, weight gain, sleep problems, and cough). [70] This study proposed candidate domains to be used to measure disease-related symptoms in this indolent disease but is not yet validated to be used in clinical trials.

CTCL is another lymphoma subtype in which QoL is of particular importance. One study showed that based on an inductive thematic analysis of 18 patients with CTCL, several domains were identified as important to CTCL patient QoL but not captured comprehensively by any existing validated QoL measure [71]. Even Skindex-29, which was used to capture symptom burden in the CTCL clinical trials described above, does not capture all domains important to CTCL patients comprehensively according to this study [23]. Another metric intended to be more disease-specific for CTCL, entitled MF/SS CTCL QoL, is also missing several important domains and was not made with patients with advanced-stage disease who have worse QoL; it has not been validated to be used for clinical decision-making or in clinical trials [72]. As QoL is taken into account along with disease response when treating patients with CTCL, a robust tool that is sensitive to change in disease status and validated in this specific patient population is an important unmet need in treating this disease. Additionally, as has been done with the MFSAF TSS v4 in myelofibrosis, QoL should be used as an endpoint in clinical trials given its importance to clinical decision-making, highlighting the need for disease-specific measures in CTCL specifically. Thus far, there is not a survey that has been validated to be used in this manner. A disease-specific PROM could also make drug development for this disease faster and more clinically relevant and impactful. These studies underscore the need for disease-specific measures even within lymphoma given the heterogeneity of the disease and the major differences in the disease natural history and treatment strategies.

## 9. Conclusions

PROMs are an essential part of evaluating the patient experience in patients with lymphoma. PROs are even more important to take into account in lymphoma subtypes with a more indolent disease course, a high symptom burden, and in which patients undergo continuous treatment. Though PROMs are increasingly used in prospective lymphoma studies, their use is often non-uniform and not used in the review process leading to approval of novel therapies in this disease due to the difficulty in their analysis. When using PROMs in study design, it is therefore important to pre-specify the analysis methods that will be employed, time points should be chosen carefully and should be clinically meaningful, and disease-specific measures should be used when possible and available validated measures exist.

## Figures and Tables

**Table 1:** Summary of lymphoma phase 2 or 3 clinical trials from 2020–2024 that used PROMs.

	Numer of Trials (N = 53)
**Lymphoma Subtype**	
Aggressive lymphoma/LBCL	16
iNHL/FL/MZL	13
NHL/B cell malignancies	5
Chronic lymphocytic leukemia/small lymphocytic lymphoma	5
Cutaneous T-cell lymphoma/mycosis fungoides/Sezary syndrome	4
Mantle cell lymphoma	4
Hodgkin lymphoma	2
Central nervous system lymphoma	1
Peripheral T-cell lymphoma	1
Primary mediastinal B-cell lymphoma	1
Waldenstrom’s macroglobulinemia	1
**Year of trial start date**	
2020	6
2021	14
2022	8
2023	14
2024	11
**Trial Endpoint**	
Primary	3
Secondary	50

**Table 2:** PROMs used in lymphoma phase 2 or 3 clinical trials from 2020–2024 [16,17,18,19,20,21,22,23,24,25,26,27,28].

PROM Used	Number of Trials (N = 53)	Domain Measured
EORTC-QLQ-C30	25	QoL
EQ-5D-5L	20	Health status
FACT-Lym	20	HRQoL
FACT-G	11	HRQoL
PRO-CTCAE	6	Symptomatic toxicity
PGI-C	6	Global impression of change
PGI-S	5	Global impression of severity
NFLymSI-18	4	Symptom burden
Skindex-29	4	QoL
EORTC QLQ CLL17	3	QoL
FACT/GOG/NTX	3	Symptom burden (peripheral neuropathy)
SF-36	2	Health status + QoL
VAS-itch	2	Symptom burden (pruritis)
FACIT	2	QoL
FACT-Leukemia	2	HRQoL
Unspecified	2	n/a

## Data Availability

Not applicable.

## Supplementary Materials

The following supporting information can be downloaded at: https://www.mdpi.com/article/10.3390/curroncol32050265/s1, Figure S1: Source: Page MJ, et al. BMJ 2021;372:n71. doi: 10.1136/bmj.n71. This work is licensed under CC BY 4.0. To view a copy of this license, visit https://creativecommons.org/licenses/by/4.0/; Table S1: PROM Website References.
