# Supplementary material for: Patient-Reported Outcome Measures (PROMS) in Lymphoma
Source: Curr Oncol. 2025 May 1;32(5):265. doi: 10.3390/curroncol32050265 (PMC12110126; doi:10.3390/curroncol32050265)
Supplement: Supplementary file 1 [file curroncol-32-00265-s001.zip › curroncol-3549483-SI.pdf]

PRISMA 2020 flow diagram for new systematic reviews which included searches of databases, registers and other sources

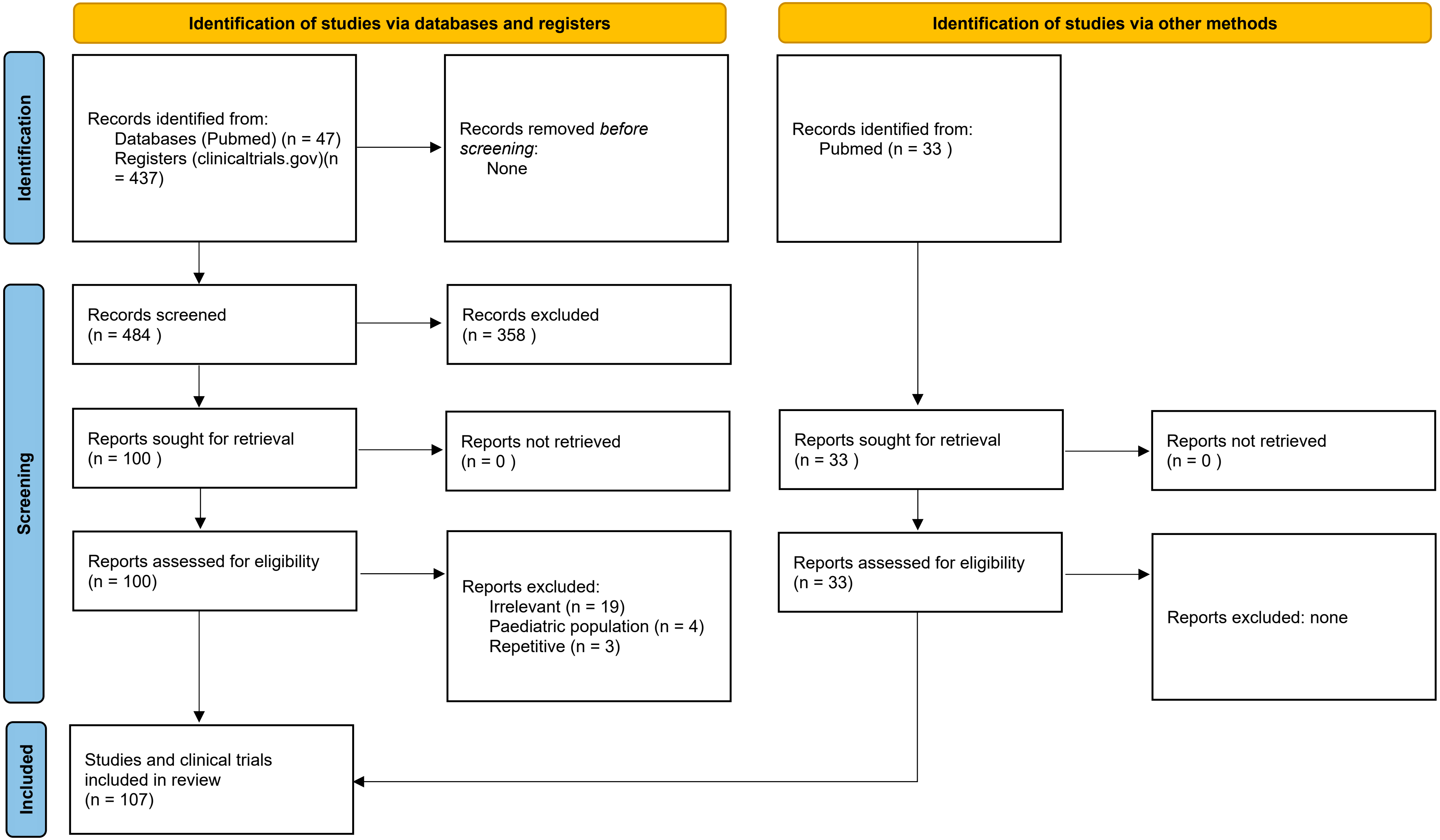

*Consider, if feasible to do so, reporting the number of records identified from each database or register searched (rather than the total number across all databases/registers).
**If automation tools were used, indicate how many records were excluded by a human and how many were excluded by automation tools.

Source: Page MJ, et al. BMJ 2021;372:n71. doi: 10.1136/bmj.n71.

This work is licensed under CC BY 4.0. To view a copy of this license, visit https://creativecommons.org/licenses/by/4.0/

PRISMA 2020 flow diagram for new systematic reviews which included searches of databases, registers and other sources

Supplemental Table S1: PROM Website References

PROM	Website
EORTC-QLQ-C30	https://www.eortc.org/app/uploads/sites/2/2018/08/Specimen-QLQ-C30-English.pdf
EQ-5D-5L	https://euroqol.org/information-and-support/euroqol-instruments/eq-5d-5l/
FACT-Lym	https://www.facit.org/measures/fact-lym
FACT-G	https://www.facit.org/measures/fact-g
PRO-CTCAE	https://healthcaredelivery.cancer.gov/pro-ctcae/
PGI-C	https://eprovide.mapi-trust.org/instruments/patient-global-impressions-scale-change-improvement-severity
PGI-S	https://eprovide.mapi-trust.org/instruments/patient-global-impressions-scale-change-improvement-severity
NFLymSI-18	https://www.facit.org/measures/nflymsi-18
Skindex-29	https://eprovide.mapi-trust.org/instruments/skindex
EORTC QLQ CLL17	https://qol.eortc.org/questionnaire/qlq-cll17/
FACT/GOG/NTX	https://www.facit.org/measures/fact-gog-ntx
SF-36	https://www.rand.org/health-care/surveys_tools/mos/36-item-short-form.html
VAS-itch	http://www.pruritussymposium.de/itchintensity.html
FACIT	https://www.facit.org/
FACT-Leukemia	https://www.facit.org/measures/fact-leu
